# The impact of cardiovascular risk factors in non-classical Fabry disease

**DOI:** 10.1186/s13023-026-04425-z

**Published:** 2026-06-04

**Authors:** Bram C. F. Veldman, Laura van Dussen, Mareen R. Datema, Rutger Meinsma, Judith Jansen-Meijer, Janet Koster, André B. P. van Kuilenburg, Maud Janssens, Lourens F. H. J. Robbers, Mirjam Langeveld

**Affiliations:** 1 Department of Endocrinology and Metabolism, Amsterdam Gastroenterology & Metabolism, Amsterdam UMC, University of Amsterdam, Amsterdam, The Netherlands; 2 Laboratory Genetic Metabolic Diseases, Department of Clinical Chemistry, Amsterdam UMC, Vrije Universiteit Amsterdam, Amsterdam, The Netherlands; 3 Department of Cardiology, Amsterdam UMC, University of Amsterdam, Amsterdam, The Netherlands; 4 Amsterdam UMC – Location AMC, Meibergdreef 9, Amsterdam, 1105 AZ The Netherlands

**Keywords:** Fabry disease, Atypical phenotype, Late-onset variant, Non-classical phenotype, Cardiovascular risk factors, LysoGb3, Cardiomyopathy

## Abstract

**Background:**

Recent advances in diagnostic and screening technologies have led to increased identification of presumed pathogenic *GLA* variants associated with non-classical Fabry disease (FD). However, the high number of identified carriers contrasts with the far lower incidence of clinical FD cases, raising questions about the clinical impact of these variants. Identifying drivers of symptom development and clinical heterogeneity is needed for guiding monitoring and intervention.

**Results:**

This study investigated a large cohort of 42 patients carrying the same p.I319T *GLA* variant to explore prognostic markers and disease-modifying factors in a monogenic context. Functional analysis of the variant in a *GLA* knockout HEK cell line revealed substantial residual enzyme activity (7%), and patients predominantly exhibited a cardiac phenotype, both consistent with non-classical FD. Among males, plasma lysoGb3 levels were markedly elevated, and all developed cardiac disease from age 50. Females showed variable clinical impact: those with intermediate plasma lysoGb3 levels developed cardiac complications after age 60, while those with low levels (< 2.3 nmol/L) rarely developed disease complications, even at older ages. Notably, 86% of the cohort had cardiovascular disease (CVD) risk factors, which likely contributed to the cardiac manifestations.

**Conclusions:**

These findings suggest that while non-classical *GLA* variants may confer genetic susceptibility to cardiac disease, clinical expression is likely strongly influenced by CVD risk factors. Patients with elevated lysoGb3 may benefit from FD-specific therapy, but strict CVD risk management remains equally important. This study underscores the importance of integrating lifestyle interventions and CVD risk factor modification in carriers of non-classical FD associated *GLA* variants.

**Supplementary Information:**

The online version contains supplementary material available at 10.1186/s13023-026-04425-z.

## Background

Fabry disease (FD; OMIM #301500) is an X-linked lysosomal storage disorder, caused by deleterious variants in the *galactosidase alpha (GLA)* gene, leading to significantly reduced α-galactosidase A (AGAL) enzyme activity and accumulation of the enzyme substrate globotriaosylceramide (Gb3) and its derivative globotriaosylsphingosine (lysoGb3). Males with (near) absent AGAL develop the classical childhood onset form of the disease, presenting with specific signs and symptoms (acroparesthesia, angiokeratoma, and cornea verticillata), followed by progressive involvement of the heart, kidneys and brain [1]. The clinical course of this classical FD is well-described with progressive loss of renal function and the development of cerebrovascular incidents in most patients, and cardiomyopathy, conduction disorders, and arrhythmias in all patients from early adulthood onwards [2]. In females carrying *GLA* variants causing classical disease in males, residual enzyme activity is higher and substrate accumulation (reflected by plasma lysoGb3 levels) often a tenfold or even lower [3]. There is also greater intraindividual variation in females, largely because of random X-chromosome inactivation [4]. As a result, the phenotype in female patients is far more variable, often limited to late-onset cardiac disease [1]. In males with ‘milder’ *GLA* variants, the phenotype - referred to as late-onset, atypical, non-classical or cardiac variant FD - resembles that of affected females with a severe *GLA* variant, but with less intraindividual variation and with plasma lysoGb3 levels in a narrower range [3]. Again, the clinical phenotype is dominated by cardiac disease, with renal and cerebrovascular involvement observed less frequently [1]. FD-associated complications occur approximately 10 years later compared to males with classical FD [5]. There is however limited knowledge on the exact impact of different ‘milder’ *GLA* variants, as well as disease modifying non-genetic factors such as comorbidities or lifestyle factors. This uncertainty is even greater for females carrying these variants, as clinical complications may not appear until late adulthood or not at all [5]. 

Over the past two decades, advances in diagnostics – particularly next-generation sequencing (NGS) - have led to a sharp rise in the identification of presumed pathogenic *GLA* variants and individuals carrying them (manuscript submitted). NGS enabled panel testing in high-risk patients presenting with signs or symptoms potentially linked to FD, such as hypertrophic cardiomyopathy or kidney disease [6, 7]. Furthermore, NGS has facilitated newborn screening (NBS) for FD in several countries [8], as well as population-level estimates of individuals carrying presumed deleterious *GLA* gene variants [9, 10]. While earlier prevalence estimates based on observed clinical cases with mostly classical FD ranged from 1 in 40.000 to 1 in 170.000 [11], NBS studies report much higher rates up to 1 in 3.000 for non-classical disease associated *GLA* variants [8], with some variants (e.g. NM_000169.3(GLA): c.640-801G > A, previously reported as IVS4 + 919G > A) in as many as 1 in 1.250 newborns [12]. Similarly, a UK biobank study in an unselected population sample found a prevalence of non-classical *GLA* variants of 1 in 5.732 [10], with comparable frequencies observed in a US population study [13]. 

The observed marked discrepancy between the frequency of presumed pathogenic *GLA* variants that are identified, and the number of observed clinical FD cases raises questions about the clinical impact of these variants. Since the presence of a presumed pathogenic *GLA* variants seems not to necessarily imply a clinical diagnosis of FD, it is essential to identify factors that influence the development of clinical signs and symptoms and explain the clinical heterogeneity, in order to guide interventions and avoid unnecessary psychosocial and healthcare burden.

This study investigates individuals carrying the p.I319T (c.956T > C) *GLA* variant, associated with non-classical FD. As the most common *GLA* variant in the Netherlands and a non-classical phenotype example, its high carrier rate enables studying prognostic markers and disease-modifying factors on a single variant background. In this way, we aim to improve prognostication for individuals identified as carrying *GLA* variants that cause AGAL insufficiency - though not a complete absence of activity. These data can be used to design strategies for monitoring and complication prevention and take a first step in identifying who may and may not benefit from FD specific therapy.

## Methods

### Participant inclusion and data collection

This study is in accordance with the Declaration of Helsinki. The study included all patients identified with the p.I319T variant in the *GLA* gene registered at the national referral center for FD in the Netherlands (Amsterdam UMC, location AMC) who provided informed consent. Two identified patients could not be included in this study, as informed consent could not be obtained. Clinical, biochemical, and functional data were collected during routine outpatient visits and prospectively entered into the Dutch Fabry Disease Register (ethics approval code: AMC2014_192). Only data during observation without FD-specific treatment (either prior to treatment or all data in those patients who never received FD-specific treatment), were included in the analyses. All patients received supportive care, including management of cardiovascular risk factors. The observation period ended when FD-specific therapy was started, when patients were no longer followed at our hospital, or at the time of closure of data collection (January 2025), whichever came first.

### Clinical data: definition and collection

At diagnosis, the presence of classical FD features - angiokeratoma, acroparesthesia and cornea verticillata – was scored. During the observation period, cardiovascular risk factors were monitored, including hypertension (defined as systolic blood pressure ≥ 140 mmHg and/or diastolic blood pressure ≥ 90 mmHg on at least two separate occasions, or based on 30-minute or 24-hour measurements, or a history of hypertension with antihypertensive drug treatment), smoking (current or former smoker), obesity (body mass index (BMI) of ≥ 30 kg/m^2^), diabetes mellitus (type 1 or 2), hypercholesterolemia (defined as untreated total- or low-density lipoprotein (LDL)-cholesterol > 95th percentile for sex and age, or a history of treated hypercholesterolemia), and chronic kidney disease (eGFR < 60 mL/min/1.73m^2^).

Cardiac assessment included plasma troponin T and NT-proBNP quantification (ng/L) and cardiac MRI (cMRI). Troponin T levels ≤ 14 ng/L were considered normal. NT-proBNP was interpreted using sex- and age-specific reference values (Additional file Table 1). Values below the laboratory’s quantification limits (< 5 ng/L for troponin T and < 50 ng/L for NT-proBNP) were included as 5 ng/L and 50 ng/L, respectively. On cMRI, left ventricular mass index (LVMI, g/m^2^) was calculated using the DuBois formula for body surface area. Left ventricular hypertrophy (LVH) was defined as a LVMI exceeding the upper bound of the 95% prediction interval based on sex- and age-specific reference values (Additional file Table 2) [14]. Myocardial fibrosis was defined by the presence of gadolinium late enhancement (GLE) on cMRI. Cardiac events during the observation period were recorded according to the definition previously described [5]. 

**Table 1:** Characteristics of included patients

		Males			Females	
	Total *n* = 17	Index cases *n* = 13	Family screening *n* = 4	Total *n* = 25	Index cases *n* = 6	Family screening *n* = 19
**Diagnosis**						
Age at diagnosis (years)	55 (20–70) *n* = 17	61 (25–70) *n* = 13	31.5 (20–55) *n* = 4	54 (14–80) *n* = 25	69.5 (63–80) *n* = 6	50 (14–72) *n* = 19
α-GAL A activity in leucocytes (% of mean reference)	1.37 (0.98–6.7) *n* = 17	1.57 (0.98–2.2) *n* = 13	1.18 (1.18–1.37) *n* = 4	66.0 (11.8–126) *n* = 25	57.2 (11.8–126) *n* = 6	66.0 (20.6–92.9) *n* = 19
Plasma lysoGb3 (nmol/L)	16.9 (9.0-35.7) *n* = 17	18.8 (9.8–35.7) *n* = 13	12.6 (9.0-15.3) *n* = 4	2.0 (0.75–5.6) *n* = 25	2.68 (2.2–5.6) *n* = 6	1.8 (0.75–2.7) *n* = 19
Genetic testing because of						
Cardiac symptoms		77%, *n* = 10			100%, *n* = 6	
Renal symptoms		23%, *n* = 3				
**Observation period**						
Age at end of observation (years)	61 (25–71) *n* = 17	64 (30–71) *n* = 13	34.5 (25–62) *n* = 4	59 (19–81) *n* = 25	74 (66–81) *n* = 6	55 (19–77) *n* = 19
Duration of observation (months/years)	3.1y (1.5 m-7.2y) *n* = 17	3.1y (1.5 m-5.4y) *n* = 13	4.4y (11.5 m-7.2y) *n* = 4	4.9y (5 m-10.9y) *n* = 25	1.6y (5 m-6.7y) *n* = 6	4.9y (10.5 m-10.9y) *n* = 19
Reason for end of observation						
Start FD-specific therapy	35%, *n =* 6	38%, *n* = 5	25%, *n =* 1	16%, *n =* 4	33%, *n =* 2	11%, *n =* 2
Transfer of care	18%, *n =* 3	15%, *n =* 2	25%, *n =* 1	12%, *n =* 3	50%, *n =* 3	
Patient’s wish	6%, *n =* 1	8%, *n =* 1		4%, *n =* 1		5%, *n =* 1
Death	12%, *n =* 2	15%, *n =* 2				
Data collection closed^a^	29%, *n* = 5	23%, *n* = 3	50%, *n =* 2	68%, *n =* 17	17%, *n =* 1	84%, *n =* 16
**Cardiac parameters**						
Elevated levels of troponin T^b^						
Yes	70%, *n* = 7	100%, *n* = 6	25%, *n* = 1	52%, *n* = 11	100%, *n* = 4	41%, *n* = 7
No	30%, *n* = 3		75%, *n* = 3	48%, *n* = 10		59%, *n* = 10
Elevated levels of NT-proBNP^c^						
Yes	70%, *n* = 7	86%, *n* = 6	25%, *n* = 1	24%, *n* = 5	75%, *n* = 3	12%, *n* = 2
No	30%, *n* = 3	14%, *n* = 1	75%, *n* = 3	76%, *n* = 16	25%, *n* = 1	88%, *n* = 15
Left ventricular hypertrophy^d^						
Yes	73%, *n* = 8	88%, *n* = 7	33%, *n* = 1	75%, *n* = 12	100%, *n* = 4	67%, *n* = 8
No	28%, *n* = 3	13%, *n* = 1	67%, *n* = 2	25%, *n* = 4		33%, *n* = 4
Myocardial fibrosis^e^						
Yes	67%, *n* = 8	78%, *n* = 7	33%, *n* = 1	64%, *n* = 14	100%, *n* = 5	53%, *n* = 9
No	33%, *n* = 4	22%, *n* = 2	67%, *n* = 2	36%, *n* = 8		47%, *n* = 8
**Renal parameters**						
Impaired renal function^f^						
Yes	19%, *n* = 3	15%, *n* = 2	33%, *n* = 1	4%, *n* = 1	20%, *n* = 1	
No	81%, *n* = 13	85%, *n* = 11	67%, *n* = 2	96%, *n* = 23	80%, *n* = 4	100%, *n* = 19
Albuminuria^g^						
Yes	69%, *n* = 11	75%, *n* = 9	50%, *n* = 2	32%, *n* = 8	67%, *n* = 4	21%, *n* = 4
No	31%, *n* = 5	25%, *n* = 3	50%, *n* = 2	68%, *n* = 17	33%, *n* = 2	79%, *n* = 15
Individuals on ACEi/ARB						
Yes	67%, *n* = 10	73%, *n* = 8	50%, *n* = 2	36%, *n* = 9	50%, *n* = 3	32%, *n* = 6
No	33%, *n* = 5	27%, *n* = 3	50%, *n* = 2	64%, *n* = 16	50%, *n* = 3	68%, *n* = 13

Note: Data are presented as medians (range) with the number of patients for whom values were available, or as percentages with denominators indicating the number of patients in the subgroup with available data

Abbreviation: FD, fabry disease; α-GAL A, alpha-galactosidase A; lysoGb3, lysoceramidetrihexoside; eGFR, estimated glomerular filtration rate; ACEi, angiotensin-converting enzyme inhibitor; ARB, angiotensin receptor blocker

^a^: Data collection was closed on 1 January 2025. ^b^: Above 14 ng/L at any point during observation. ^c^: Above the sex- and age-specific upper limit of normal at any point during observation. ^d^: A left ventricular mass indexed for body surface area exceeding the upper bound of the 95% prediction interval based on sex- and age-specific reference values at any point during observation. ^e^: Defined by the presence of gadolinium late enhancement on cardiac magnetic resonance imaging at any point during observation. ^f^: An estimated glomerular filtration rate using the CKD-EPI 2021 formula below the sex- and age-specific lower limit of normal, at any point during observation. ^g^: Above 3.0 mg/mmol at any point during observation

Renal assessments included the estimated glomerular filtration rate (eGFR, ml/min/1.73m^2^, using the CKD-EPI 2021 formula) with normal values defined as the 2.5th to 97.5th percentile of the reference range per age group (Additional file Table 3)[15] and albuminuria defined as an albumin-to-creatinine ratio (ACR) > 3.0 mg/mmol in 24-hour or spot urine samples. Chronic kidney disease was classified based on eGFR and albuminuria severity, according to the Kidney Disease: Improving Global Outcomes (KDIGO) classification.

Cerebrovascular assessment included cerebral MRI to screen for white matter lesions (WML), cerebral infarctions, and intercranial hemorrhages. Neurological events during the observation period were recorded as transient ischemic attacks (TIAs) or cerebrovascular accidents (CVAs; ischemic or hemorrhagic).

### Family screening

Family counseling was offered to all patients. In addition, genealogical analysis using family history and publicly available records (birth, marriage, and death certificates) to establish family links was performed.

### Statistical analysis

Data were stratified by sex and method of diagnosis (index case or identification through family screening). Patients’ characteristics, clinical features, risk factors and events, were expressed as a percentage of the total number or patients within each stratified group, for whom the relevant data were available. Continuous variables were reported as medians with ranges because of the non-normal distribution.

### Genetic analysis, enzyme activity measurement and plasma lysoGb3 measurements

Genetic analysis and enzyme activity were assessed at diagnosis in all individuals. Genetic testing of the *GLA* gene was performed on EDTA whole blood using Sanger sequencing or NGS. AGAL enzyme activity (nmol/mg/h) was measured in leucocytes using a standard 4-MU assay and expressed as a percentage of the mean reference range to allow comparison across different reference values [16]. One individual’s enzyme activity was excluded from analysis because the measurement was performed in a different laboratory.

Plasma lysoGb3 (nmol/L) levels were measured at diagnosis and during the observation period using tandem mass spectrometry [17], with values < 0.5 nmol/L considered normal. For each individual, the plasma lysoGb3 level used in analyses was the mean of all treatment-naïve measurements. For visualization, these values were categorized into groups, as previously described in an earlier study by our group; 0.5–2.3 nmol/L (found in females with non-deleterious *GLA* variants), 2.3–7.3 nmol/L (lower range males with non-deleterious *GLA* variants or lower range females with classical FD), 7.3–40 nmol/L (higher range males with non-deleterious *GLA* variants or higher range females with classical FD) and > 40 nmol/L (males with classical FD, not observed in this study) [3]. 

### Expression of the variant in a GLA knockout HEK cell line and dot blot analysis

*GLA* cDNA (refseq nm_000169.3) was cloned in pcDNA3.1Zeo(+) as a HindIII/XhoI fragment containing a C-terminal V5 and his tag (Genscript). The c.956T > C (p.I319T) variant was introduced using site-directed mutagenesis (QuikChange™ Site-Directed Mutagenesis Kit, Agilent).

HEK Flp-In *GLA* knockout cells were generated by using CRISPR-Cas9 as described by Ran et al. [18]. Therefore, guide 5’-GCTAGCTGGCGAATCCCATG-3’ targeting exon 2 is cloned in the PX458 vector (Addgene plasmid ID: 48138). HEKFlpIn cells (Invitrogen) are transfected by using jetPRIME (Polyplus transfection) after which the cells are sorted by FACS (Sony sh800) on GFP expression and plated 1 cell per well in 96 wells plate. After 3 weeks, DNA of the clones were isolated using Phire Animal Tissue Direct PCR Kit (ThermoFisher). Exon 2 is amplified with gene specific primers (5’-TTACTACCACACTATTACTGGG-3’and 5’-ACTCTTGACCTCAGGTGATC-3’) which are tagged with M13 sequences (5’-TGTAAAACGACGGCCAGT-3’ and 5’- CAGGAAACAGCTATGACC-3’). The PCR-products were sequenced using the M13 sequences with the Big DyeTM Terminator v.3.1 Cycle Sequencing Kit on an ABI 3730 sequencer (Applied Biosystems).

HEK Flp-In *GLA* knockout cells were cultured in Dulbecco’s modified eagle’s medium with 4.5 g/L glucose, 25 mM Hepes and 584 mg/L L-glutamine, supplemented with 10% fetal bovine serum, 100 U/ml penicillin, 100 mg/ml streptomycin and 250 µg/ml fungizone (Gibco) at 37 °C in a humidified 5% CO_2_ incubator. For transient transfections, cell cultures were set up in 12-well plates one day prior to transfection. Cells were transfected with pcDNA3.1Zeo-gla-v5-his (wild-type, variant or empty vector) using X-treme GENE HP DNA Transfection reagent (Merck). All transfections were performed in triplicate. Two days after transfection, cells were harvested and washed with PBS. Pellets were immediately used or stored at − 20 °C until use.

The AGAL activity of recombinantly-expressed wild-type and mutant AGAL in HEK Flp-In *GLA* knockout cells was normalized by the relative amount of AGAL as measured by dot blot. Cell supernatants (several different protein concentrations) were spotted onto nitrocellulose membrane with Bio-Dot Apparatus (Bio-Rad). The membranes were blocked using Intercept^®^ blocking buffer (LI-COR) and incubated with a *GLA* antibody produced in rabbit (Merck, SAB1410536) and a mouse anti α-tubuline/β-actine antibody (Merck T6199, A5441) in 50% Intercept^®^ blocking buffer, 50% PBS and 0.1% Tween20. Membranes were washed three times in PBS with 0.1% Tween20 and then incubated with IRDye^®^ 800CW Goat and IRDye^®^ 680RD Donkey (LI-COR) secondary antibodies, in the same blocking buffer as used for primary antibodies. Membranes were washed three times in PBS with 0.1% Tween20 scanned and dot intensities analyzed using the LI-COR Odyssey infrared imaging system. The dot intensities of AGAL were normalized for the corresponding signal of α-tubulin.

## Results

The total cohort comprised 42 patients (17 males, 25 females) with a pathogenic p.I319T variant in the *GLA* gene. Nineteen patients were index cases, primarily presenting with cardiomyopathy (16/19, 84%), and 3 with renal insufficiency (3/19, 16%)(see Table 1). Index cases were mostly male (13/19, 68%) and the male index cases were notably younger than female index cases (median age 61 (25–70) vs. 69.5 (63–80) years, respectively). The remaining 23 patients were identified through family screening, typically diagnosed two to three decades earlier compared to the index cases. Pedigree analysis of the index cases revealed an 11-generation family tree tracing (most likely) all patients to a common ancestor from the early 18th century. Based on the X-linked inheritance pattern, the linkage of the 42 patients yielded a total of 119 obligate carriers.

None of the 42 patients showed classical FD features. No angiokeratoma were observed, and cornea verticillata was absent when assessed (0/16). The presence of acroparesthesia was considered in 3 patients, but further testing ruled out small fiber neuropathy (temperature discrimination within normal limits) or identified an alternative cause for pain (carpal tunnel syndrome). In males whose AGAL enzyme activity could be quantified, the median enzyme activity was 1.3% of the mean reference range. In 6 males the enzyme activity was below the detection limit. The p.I319T variant was expressed in HEK Flp-In *GLA* knockout cells and in this cell system the AGAL activity was 7% of the activity measured in cell lines expressing the wild-type variant. Plasma lysoGb3 levels were above the upper limit of normal (> 0.5 nmol/L) in all patients. Male patients had a median plasma lysoGb3 level of 16.9 (9.0-35.7) nmol/L, slightly higher in index cases than in those identified through family screening. Females had substantially lower levels (median 2.0 (0.8–5.6) nmol/L), also slightly higher in index cases (Table 1).

The median treatment-naive observation period from diagnosis was 3 years (1.5 months-7.2 years) for males and nearly 5 years (5 months-10.9 years) for females. This observation period was relatively shorter in index cases compared to patients identified through family screening, as index cases more frequently received FD-specific therapy and sooner after diagnosis. The observation perioded ended for 10 patients (6 males, 4 females) because of treatment initiation (9 started enzyme replacement therapy, 1 started migalastat), for 6 due to transfer of care, for 2 patients at their own request, and for 2 due to death. All other patients were observed until the time of data collection (Jan 2025).

Elevated plasma troponin T levels were observed in 70% of male patients (7/10) and 52% of female patients (11/21). NT-proBNP levels were elevated in 70% of male patients (7/10) and 24% of female patients (5/21). Higher elevations of both cardiac biomarkers were predominantly noted among index cases. LVH and myocardial fibrosis were present in the majority of the index cases and in females identified through family screening. The biochemical elevations and structural abnormalities were mostly absent in male patients identified through family screening, who were generally younger.

**Fig. 1:**
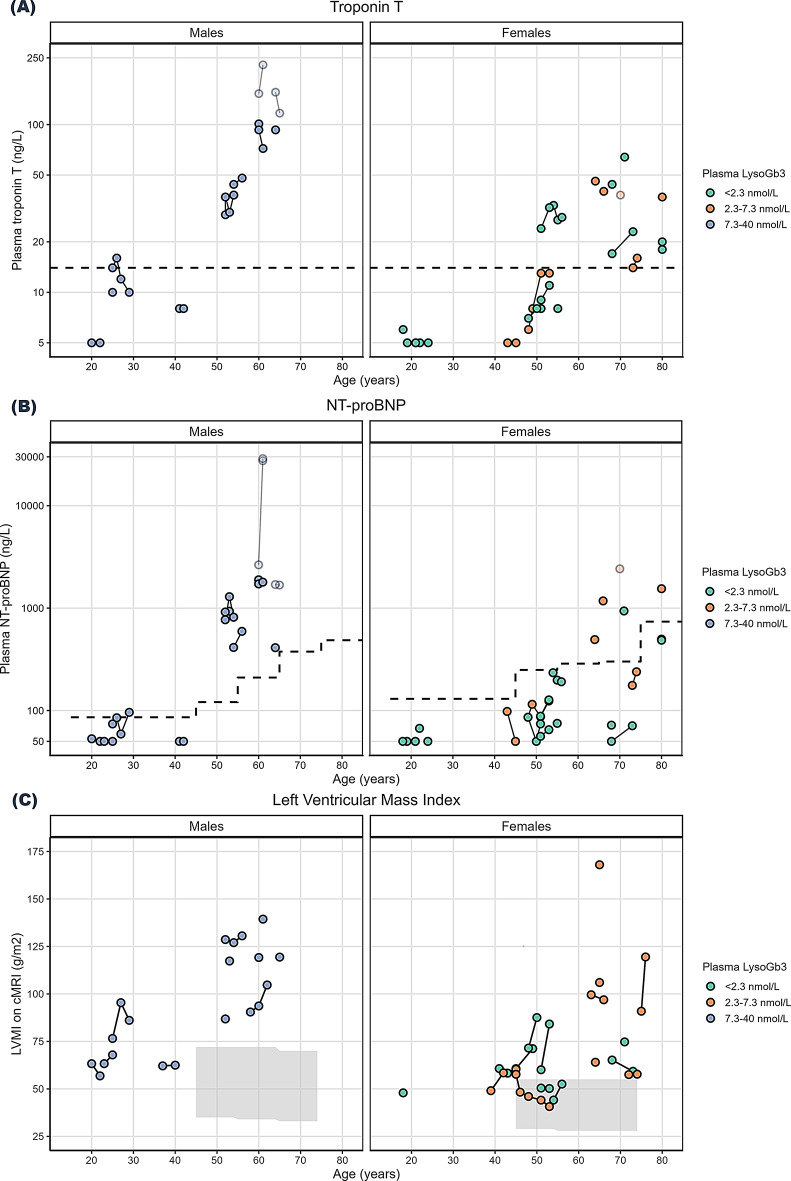
Cardiac disease markers in patients carrying the p.I319T *GLA* variant. Repeated measurements from individual patients are connected by solid lines. (**A**) Plasma troponin T levels (*n* = 31, 10 males/21 females) and (**B**) Plasma NT-proBNP levels (*n* = 32, 11 males/21 females), both shown on a logarithmic scale. Dashed lines indicate the upper limit of normal as defined by the local laboratory. Faded data points represent measurements from patients with impaired renal function, which may have a confounding effect on the levels of the cardiac biomarker. (**C**) Left ventricular mass indexed (LVMI) to body mass (*n* = 27, 11 males/16 females). The shaded area represents the lower and upper bounds of the age- and sex-specific 95% prediction interval, adapted from Petersen et al. 2017 [14]

The earliest onset and most pronounced elevations in markers of cardiac disease - troponin T, NT-proBNP, and LVMI - were observed in male patients who all had a plasma lysoGb3 levels of > 7.3 nmol/L (Fig. 1). All male patients aged over 50 years had a plasma troponin T level of > 20 ng/L and those over the age of 60 of > 50 ng/L. In females these markers of cardiac disease were far more variable and could remain (near) normal up to age 80 years old.

**Fig. 2:**
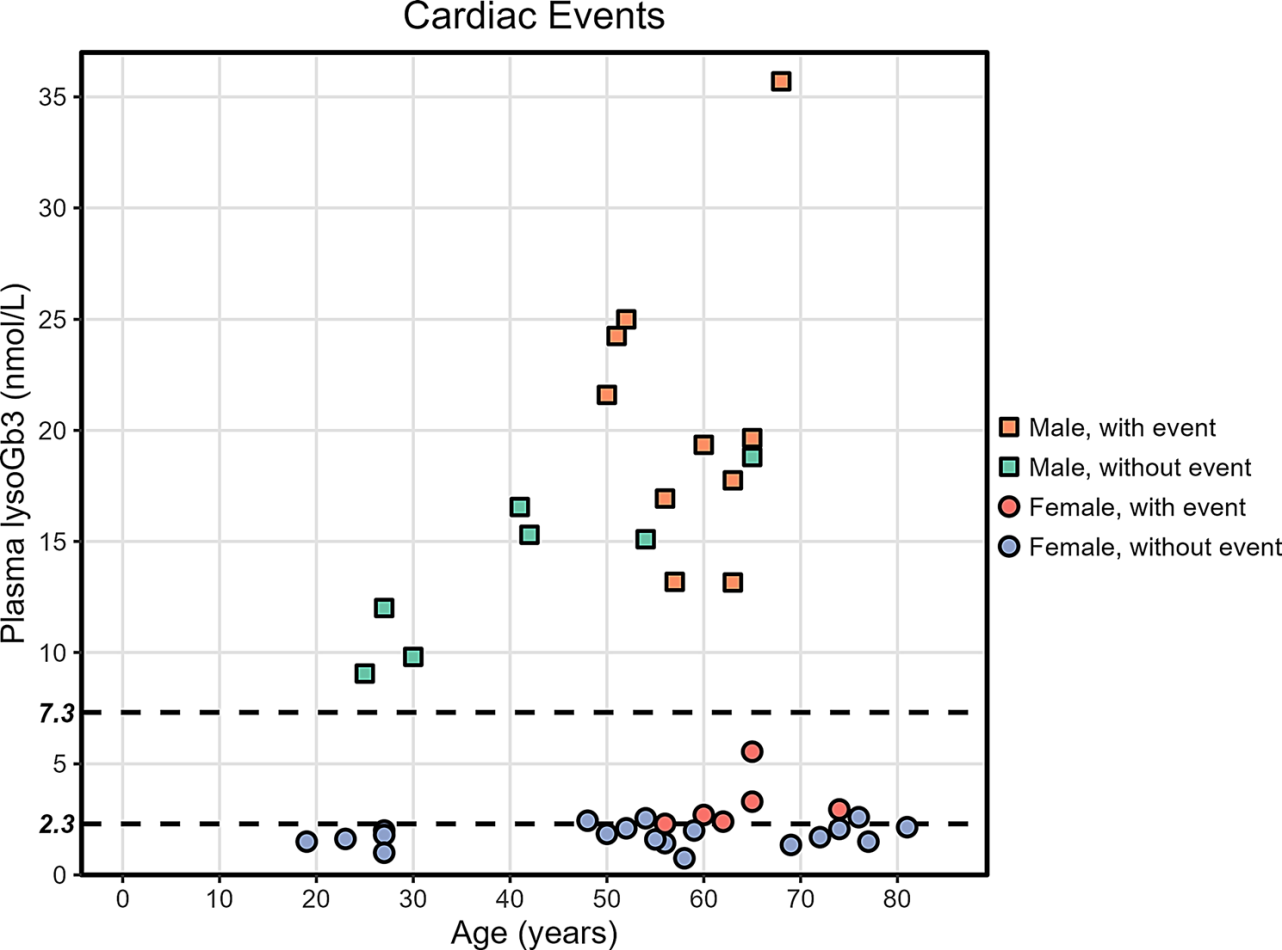
Cardiac events in patients carrying the p.I319T *GLA* variant (*n* = 16, 10 males/6 females). The y-axis shows the patient’s untreated plasma lysoGb3 level, while the x-axis indicates age at the time of the first cardiac event. For patients without cardiac events (*n* = 26, 7 males/19 females), the x-axis reflects age at last observation. Dashed lines represent plasma lysoGb3 thresholds of 2.3 and 7.3 nmol/L, which were used to categorize patients by sex and disease phenotype, as described by Van der Veen et al. 2023 [3]

A total of 16 patients (10 males, 6 females) suffered from a cardiac events during the observation period (see Table 2). The first cardiac event in males occurred at age 50, with 91% of males above this age experiencing at least one event (see Fig. 2). In females, the first event occurred at age 56, affecting 32% of those aged 50 and older, and 45% of aged 60 years and above. No cardiac events were observed in females with a plasma lysoGb3 below 2.3 nmol/L. Cardiac events were more frequent among index cases, who tended to be older; they accounted for 87.5% of all events, with 74% of index patients experiencing at least one event.

Regarding neurological events, two male index patients in this cohort experienced a CVA (both ischemic), one at age 32 and one at 61; the latter also had a TIA at age 64. WML were present in most patients; 7 of 11 (64%) males and 13 of 19 (68%) females.

**Table 2:** Occurrence of cardiac events

Cardiac events	Total *n* = 16	Males *n* = 10	Females *n* = 6
ICD/Pacemaker implantation	56%, *n* = 9	60%, *n* = 6	
Arrhythmia	50%, *n* = 8	60%, *n* = 6	33%, *n* = 2
Valvular disease	31%, *n* = 5	30%, *n* = 3	33%, *n* = 2
Myocardial infarction	19%, *n* = 3	20%, *n* = 2	17%, *n* = 1
Heart failure	19%, *n* = 3	20%, *n* = 2	17%, *n* = 1
Systolic dysfunction on cMRI	19%, *n* = 3	30%, *n* = 3	
Left ventricular outflow tract obstruction	13%, *n* = 2		33%, *n* = 2
Cardiac death	13%, *n* = 2	20%, *n* = 2	
Percutaneous coronary intervention	13%, *n* = 2	10%, *n* = 1	17%, *n* = 1
Coronary artery bypass grafting	6%, *n* = 1		17%, *n* = 1

Note: Data are presented as percentages with denominators indicating the number of patients in the subgroup. 75% (6/8) of patients with arrhythmia received an ICD/pacemaker. Both patients who underwent PCI had also experienced a myocardial infarction, and one of them additionally underwent CABG

Abbreviation: ICD, implantable cardioverter defibrillator; cMRI, cardiac MRI

**Fig. 3:**
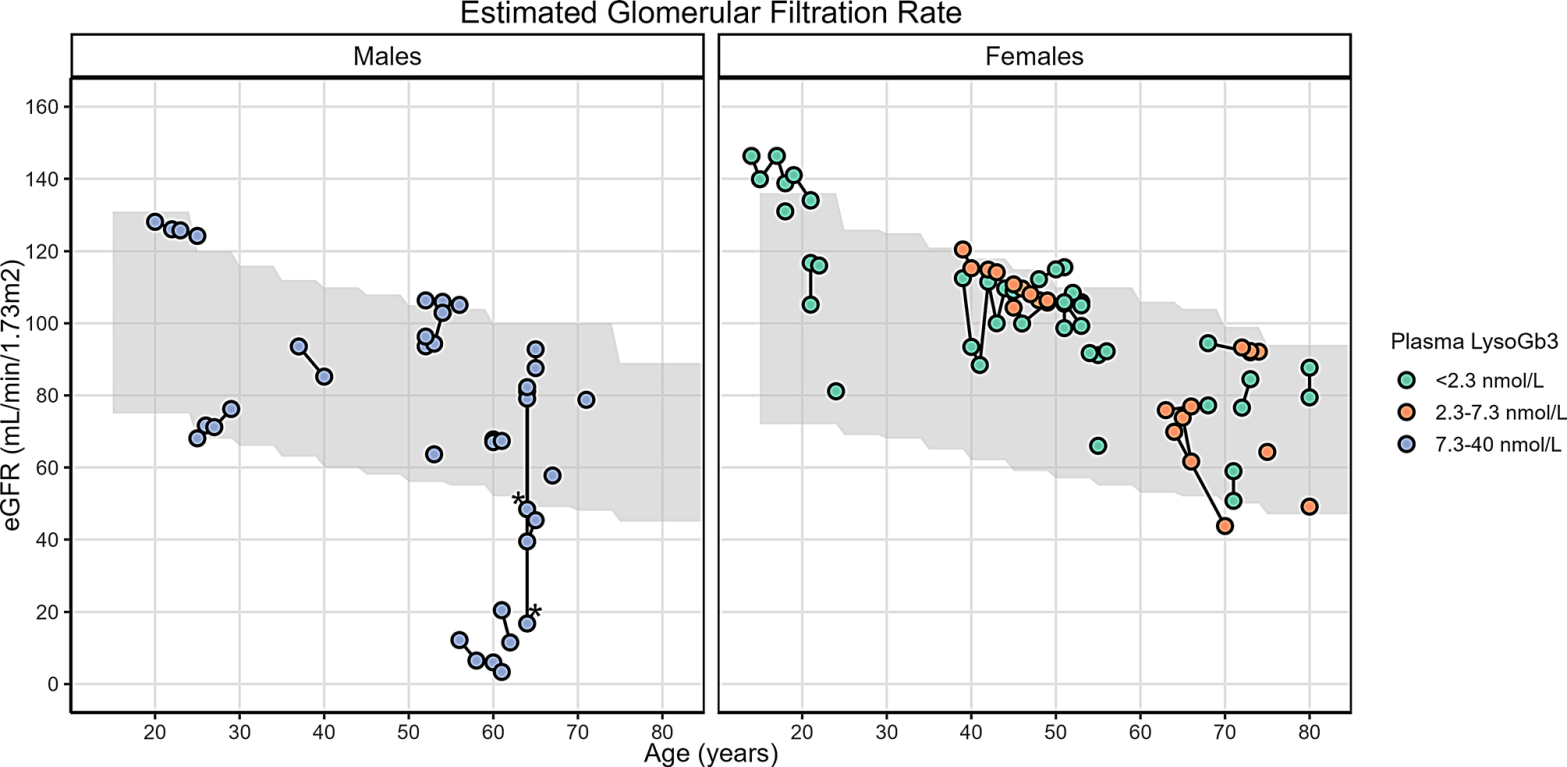
eGFR in patients carrying the p.I319T *GLA* variant (*n* = 41, 16 males/25 females). Repeated measurements from individual patients are connected by solid lines. Asterisks indicate two values from the same male patient that were artifactually lowered due to elevated plasma creatinine following cardiogenic shock. The shaded area represents 2.5th to 97.5th percentile of sex- and age-specific reference limits, adapted from Baba et al. 2015 [15]

Regarding renal status, stage 4 CKD (eGFR < 30 mL/min/1.73m^2^) was found in one male index case and one male identified through family screening (aged 61 and 56, respectively) at diagnosis. They both progressed to kidney failure during the observation period and required renal replacement therapy. In another younger index male (aged 24), FD diagnosis followed the development of end-stage renal disease; he had undergone kidney transplantation prior to diagnosis, after which his renal function recovered (CKD G2A2, eGFR ~ 70 mL/min/1.73m^2^). A moderately reduced eGFR (39–45 mL/min/1.73m^2^) was observed in a 64-year-old index male, though this was influenced by longstanding hypertension and cardiac medication. In one male index case, eGFR was low at the time of diagnosis due to temporarily elevated plasma creatinine following cardiogenic shock. Of the female patients, only one developed an eGFR below the lower age- and sex- specific reference limit during observation, attributed to hemodynamic consequences of heart failure and heart failure treatment. All other patients had an eGFR within the 2.5th to 97.5th percentile of the age- and sex-specific reference range (see Fig. 3). Albuminuria was primarily seen in index cases (13/18, 72%), and primarily in male patients. The majority of them received renoprotective therapy (angiotensin-converting enzyme inhibitors or angiotensin II receptor blockers).

**Fig. 4:**
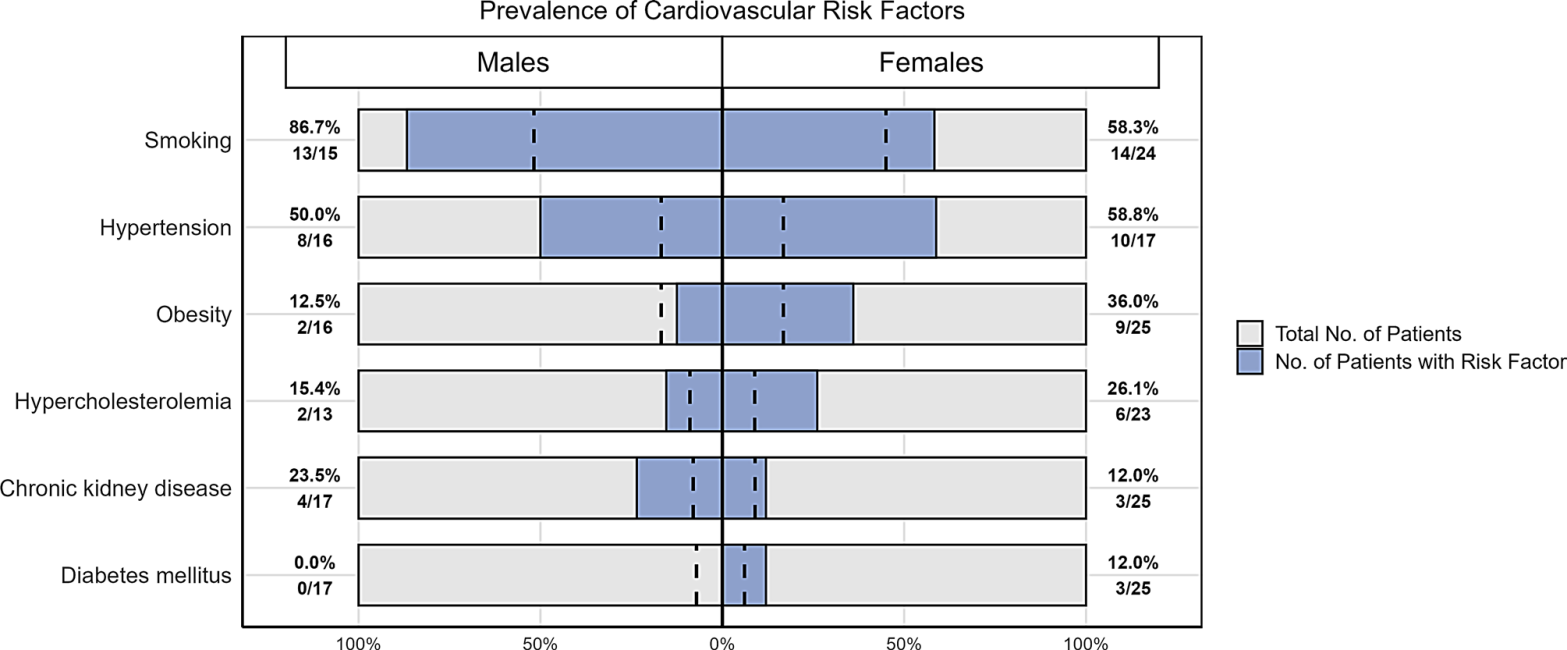
Prevalence of cardiovascular risk factors in patients carrying the p.I319T *GLA* variant, stratified per sex. Light grey horizontal bars represent the total number of patients with available data for each risk factors; blue bars indicate the proportion of patients exhibiting the respective risk factor. Percentages and absolute numbers are shown alongside the bars. Vertical dashed lines represent the prevalence of each risk factor in the general Dutch population [19–24]

**Fig. 5:**
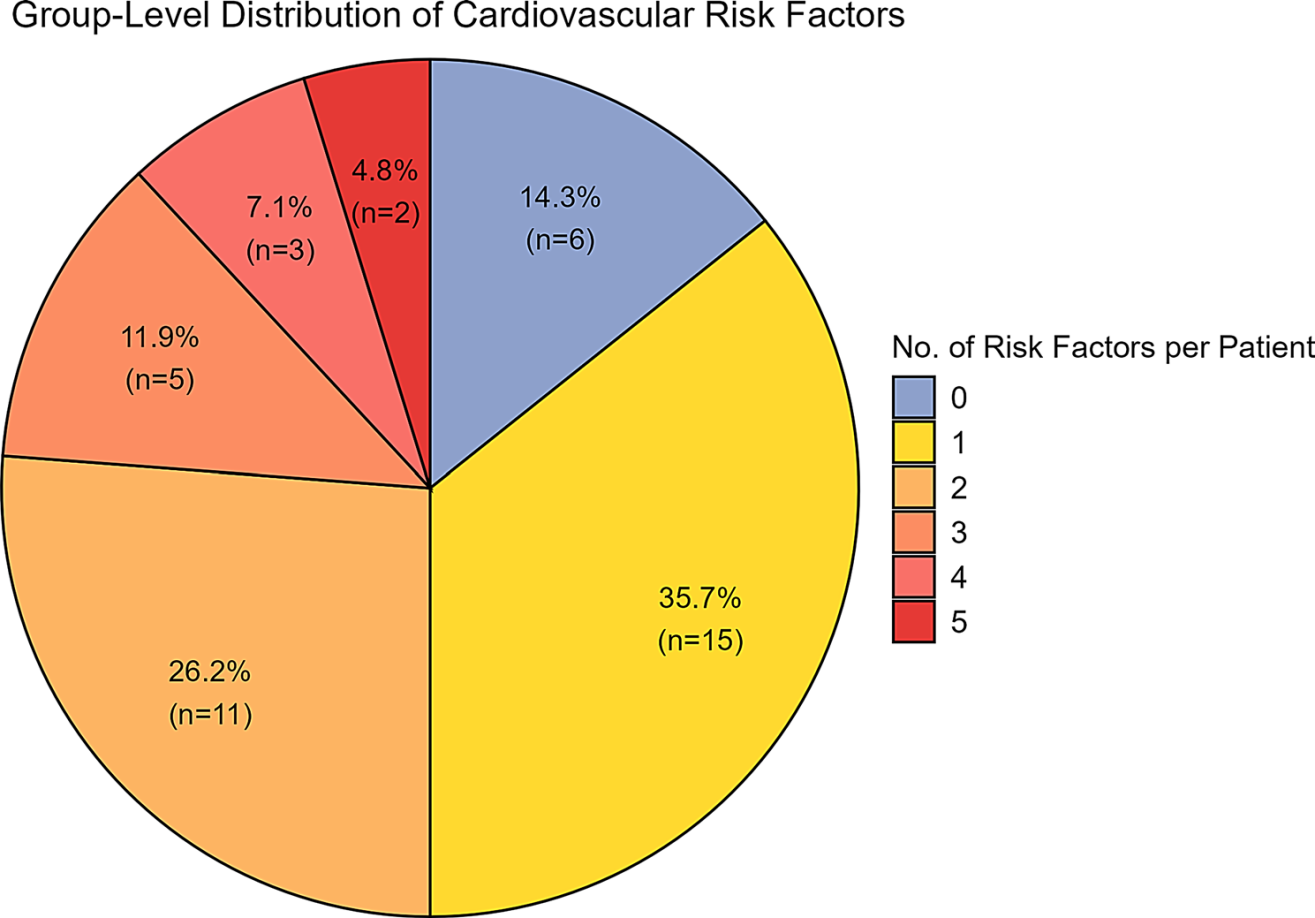
Distribution of the number of cardiovascular risk factors among patients carrying the p.I319T *GLA* variant. Each segment represents the proportion of patients with the corresponding number of risk factors

Among patients with available cardiovascular risk data, 86% (36/42) had at least one cardiovascular disease (CVD) risk factor and most patients had multiple risk factors (Figs. 4 and 5). Hypertension and a history of smoking were the most prevalent CVD risk factors. In 4 patients with hypercholesterolemia for whom pre-statin treatment cholesterol data were available, total cholesterol ranged from 7.1 to 8.6 mmol/L. In 2 cases, LDL levels also exceeded the 95th percentile for sex and age (5.1–6.2 mmol/L). Familial hypercholesterolemia was considered in one patient but ruled out.

## Discussion

This study describes the disease course in a relatively large cohort of patients carrying the p.I319T variant, a *GLA* variant associated with what is so far described as non-classical FD. Indeed, in male patients carrying this variant, the pathognomonic features of classical FD are lacking and the plasma lysoGb3 elevations are lower compared to classical FD, compatible with the non-classical disease phenotype [3]. Importantly, enzyme activity measurement in leucocytes was not helpful to distinguish this specific phenotype from classical disease, since most values were either below the limit of detection or around 1% of the mean reference, the level associated with classical disease [1]. However, expressing the variant in a *GLA* knockout HEK cell line revealed substantial residual AGAL activity (7%), which is in line with the plasma lysoGb3 level and the observed phenotype. Thus, measuring AGAL activity of recombinantly-expressed GLA enzymes in a *GLA* knockout HEK cell line can assist in classifying *GLA* variants.

Several observations can be made regarding the clinical phenotype in carriers of the p.I319T variant. First, the disease course in male patients seems quite homogeneous, with a predominant cardiac phenotype occurring in virtually all males from age 50 onwards. However, it is very important to take into account that 87% of these males were either a past or a current smoker and the incidence of other cardiovascular risk factors was also high (Fig. 4). Thus, the observed cardiac disease and cerebrovascular disease is not only the effect of the genetic predisposition resulting from carrying the *GLA* variant, but most likely also of lifestyle and comorbid factors. The relative contribution of the *GLA* variant and the more common CVD risk factors cannot be determined because of the limited sample size. Secondly, clinically significant renal disease was not observed in the majority of the current cohort and other non-classical FD cohorts [25–27], with the exception of 3 patients. In the youngest of these 3 patients with end stage renal disease, another genetic predisposition was found likely (but not identified) since there were first degree family members with early onset hypertension and renal failure not compatible with an X-linked inheritance pattern. A second renal pathology was also suspected in the oldest patient with end-stage renal disease, again unconfirmed. Thirdly, the picture for females carrying these non-classical *GLA* variants shows variable clinical impact, with a relation with the observed plasma lysoGb3 level. Overall, those females with the variant but with low levels of plasma lysoGb3 (< 2.3 nmol/L) do not develop disease complications well into older age (Fig. 2). Females with intermediate plasma lysoGb3 levels (2.3–7.3 nmol/L) can develop cardiac disease complications but rarely do so before the age of 60 (Fig. 2). Again, as in males, the prevalence of CVD risk factors was very high (88% having at least one and 48% of females more than one), indicating interplay between genetics and other factors in the development of cardiac complications.

In both males and female FD patients in this study, the prevalence of conventional CVD risk factors far exceeds rates in the general population [19–24]. This elevated risk profile is reflected in the clinical outcomes: among individuals over 50 years of age carrying the p.I319T variant who suffered a cardiac event, 88% had at least one such risk factor. The observed events - such as arrhythmias, heart failure, and myocardial infarction- are not specific to FD but also independently linked to CVD risk factors such as hypertension and smoking. In FD, cardiovascular complication risk is thought to be primarily caused by glycosphingolipid accumulation in cardiomyocytes, the conduction system and endothelium of both large and small blood vessels, resulting in hypertrophic cardiomyopathy, arrhythmia, and macro- and microvascular disease [28, 29]. Conventional CVD risk factors contribute to macrovascular disease [30, 31], but also cause inflammation and oxidative stress in the microvasculature [32]. Thus, there is a complex interplay between *GLA* insufficiency and other factors contributing to the overall risk of CVD, summarized in Table 3.

**Table 3:** Fabry disease-related manifestations and common cardiovascular events influenced by additional cardiovascular risk factors

Risk factors	LVH	Cardiac fibrosis	WML	AF	MI	Valvular disease
Hypertension	**+**(33, 34)	**+**(35)	**+**(36)	**+**(37)	**+**(38)	**+**(39)
Diabetes mellitus	**+**(33)	**+**(35)	**+**(36)	**+**(37)	**+**(38)	**+**(39)
Smoking			**+**(36)	**+**(37)	**+**(38)	**+**(39)
Obesity	**+**(33, 34)	**+**(35)		**+**(37)	**+**(38)	
Hypercholesterolemia					**+**(38)	**+**(39)
Fabry disease	**+**(40, 41)	**+**(40)	**+**(42)	**+**(43)	**+**(44)	**+**(45)

Note: A plus symbol indicates that the specific factor independently drives the disease mechanism that is described to lead to complications in Fabry disease [33–45]. Only factors considered independent contributors are listed, acknowledging that many additional indirect associations may also exist. This list is not exhaustive. Co-occurrence of several factors may amplify the overall risk of specific endpoints through distinct mechanisms. For example, *GLA* insufficiency (glycosphingolipid accumulation) + smoking (proinflammatory effects) + hypertension (structural remodeling) = cumulatively increased risk of atrial fibrillation. Similarly, *GLA* insufficiency (glycosphingolipid accumulation) + diabetes (proinflammatory effects) + hypercholesterolemia (atherosclerotic plaque formation) = cumulatively increased risk of myocardial infarction

Abbreviation: LVH, left ventricular hypertrophy; WML, white matter lesions; AF, atrial fibrillation; MI, myocardial infarction

**Fig. 6:**
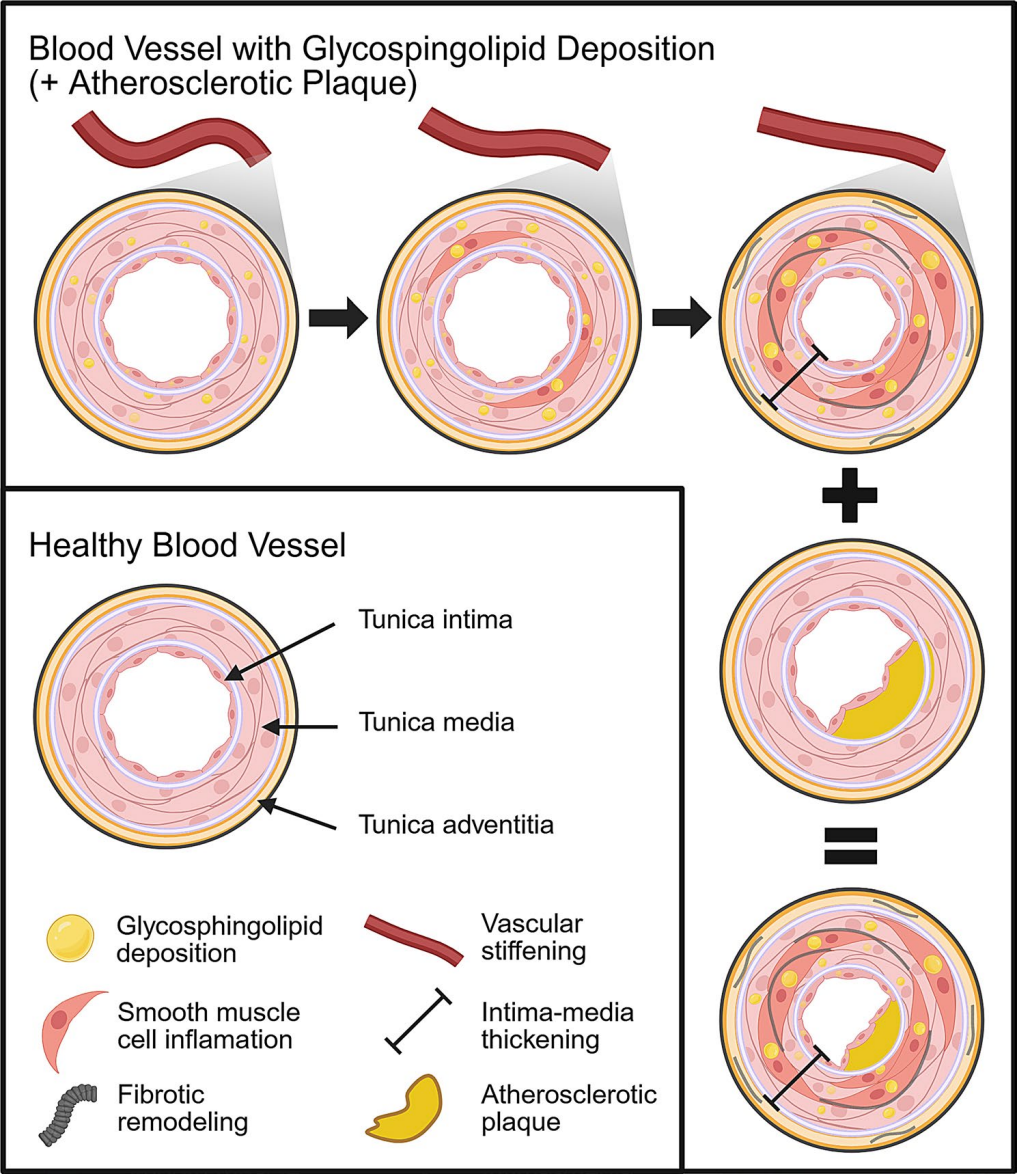
Potential mechanisms in the development of Fabry disease-related vasculopathy and the interplay with common atherosclerosis. Intracellular accumulation of globotriaosylceramide (Gb3) and lysoceramidetrihexoside (lysoGb3) leads to upregulation of adhesion molecules, reduced nitric oxide synthesis, and increased reactive oxygen species production. These changes promote oxidative stress, inflammation, and cellular injury, impairing vasodilatory function, and vascular remodeling [28, 46, 47]. In non-classical FD, smooth muscle cell involvement appears more prominent, characterized by proliferation and hypertrophy, which likely precedes endothelial dysfunction [47]. The resulting structural alterations - including intima-media thickening, subendothelial and extracellular matrix remodeling, and fibrotic changes – reduce vascular elasticity and enhance hypercontractility. Ultimately, these changes culminate in vascular stiffening, luminal narrowing, and progressive vascular damage. According to our hypothesis, the coexistence of conventional risk factors, such as atherosclerosis, further amplifies the risk of cardiovascular complications through their interaction and cumulative effects with sphingolipid accumulation

Figure 6 is an illustration of how this interplay could affect the arterial vessel wall, ultimately leading to systemic vascular wall disease, which has been described in FD patients (studies summarized in the review by Faro et al.)[28]. These independently contributing factors result in an increased risk of micro- and macrovascular disease and we suggest patients with combined *GLA* insufficiency should be offered monitoring of the vascular status as well as strict treatment of comorbidities such as hypercholesterolemia, hypertension and obesity, with a lower threshold for intervention compared to the general population.

To summarize, based on the current findings in this cohort and data from previous studies [46, 48, 49], we propose the following model: in individuals carrying non-classical *GLA* variants that are associated with AGAL insufficiency rather than deficiency the *GLA* variant acts as a genetic risk factor, contributing to the overall risk of developing CVD. In addition to the genetic risk, conventional CVD risk factors can serve as a second hit, triggering disease expression in the genetically predisposed individuals – a mechanism similarly observed for *APOL1* variants, which increase CKD’s risk but require co-occurring factors for disease onset [50]. Measuring plasma lysoGb3 can help to estimate the weight of the *GLA* variant-associated risk, with a higher risk if plasma lysoGb3 levels are more prominently elevated. The estimated relative impact of the genetic variant should also direct the follow-up and treatment approach for carriers of *GLA* variants, associated with non-classical FD. In individuals with a low plasma lysoGb3 level (< 2.3 nmol/L in our center), no FD-specific therapy should be initiated, and one should primarily counsel the patients towards a healthy lifestyle. High frequency stringent monitoring is not necessary. In individuals with higher lysoGb3 levels, the first step should be aggressive identification and modification of conventional CVD risk factors: stimulating physical activity, early smoking prevention/help with cessation, low sodium intake, maintaining systolic blood pressure < 130 mmHg if tolerated, LDL cholesterol < 1.4 mmol/L, avoiding overweight, tight diabetes control and renal protective interventions if indicated [51]. Subsequently, a monitoring plan for the early discovery of FD-specific manifestations should be put in place and FD-specific therapy should be initiated if these occur. In individuals with markedly higher plasma lysoGb3 levels FD-specific therapy can be considered earlier on, though strict CVD risk management remains equally important.

It is important to note that the hypothesis regarding the interplay between the genetic risk and conventional risk factors, along with the proposed monitoring model, should be tested in other cohorts of individuals with *GLA* insufficiency. This would help confirm our findings and further refine diagnostic, follow-up, and treatment strategies. There is a growing body of evidence showing the association between CV risk factors and their impact on the phenotype in the presence of *GLA* variants. Recently, Giammanco et al. demonstrated that carriers of *GLA* variants of uncertain pathogenicity, were only associated with the FD phenotype if the CV risk profile was elevated [52]. In addition, a study on real-world clinical outcomes in FD found that in patients carrying non-classical *GLA* variants with poorer clinical outcomes more CVD risk factors were present [53]. The authors concluded that these risk factors likely influenced clinical outcomes and contributed to an elevated risk of CVD complications and mortality, emphasizing the importance of systematic assessment and management.

## Conclusions

This study proposes a model providing practical guidance on identifying individuals at risk and implementing targeted interventions, representing an important step forward in improving outcomes for those with *GLA* insufficiency. At the same time, it is important to move away from the current practice of labeling all individuals carrying a *GLA* variant that impacts AGAL activity as FD patients, as this leads to unnecessary monitoring and disease-specific treatment. This poses a burden on both these individuals and society.

## Supplementary Information

Below is the link to the electronic supplementary material.

Supplementary Material 1

## Data Availability

The datasets used and/or analysed during the current study are available from the corresponding author on reasonable request.

## Abbreviations

AGAL: α-Galactosidase A
ACR: Albumin-to-Creatinine Ratio
APOL1: Apolipoprotein L1
BMI: Body Mass Index
cDNA: Copy Deoxyribonucleic Acid
cMRI: Cardiac Magnetic Resonance Imaging
CKD: Chronic Kidney Disease
CVA: Cerebrovascular Accident
CVD: Cardiovascular Disease
DNA: Deoxyribonucleic Acid
EDTA: Ethylenediaminetetraacetic Acid
eGFR: Estimated Glomerular Filtration Rate
FACS: Fluorescence-Activated Cell Sorting
FD: Fabry Disease
Gb3: Globotriaosylceramide
GFP: Green Fluorescent Protein
GLA: Galactosidase Alpha
GLE: Gadolinium Late Enhancement
HEK: HEK 293 cells
KDIGO: Kidney Disease Improving Global Outcomes
LDL: Low-Density Lipoprotein
LVH: Left Ventricular Hypertrophy
LVMI: Left Ventricular Mass Index
LysoGb3: Glotriaosylsphingosine
NBS: Newborn Screening
NGS: Next-Generation Sequencing
NT-proBNP: N-terminal Prohormone of Brain Natriuretic Peptide
PBS: Phosphate Buffered Saline
PCR: Polymerase Chain Reaction
TIA: Transient Ischemic Attack
UK: United Kingdom
US: United States of America
WML: White Matter Lesions
4-MU: 4-Methylumbelliferone
