# Supplementary material for: The impact of cardiovascular risk factors in non-classical Fabry disease
Source: Orphanet J Rare Dis. 2026 Jun 4;21:275. doi: 10.1186/s13023-026-04425-z (PMC13455293; doi:10.1186/s13023-026-04425-z)
Supplement: Supplementary file 1 [file 13023_2026_4425_MOESM1_ESM.docx]

**SUPPLEMENTAL TABLE 1 | Age- and sex-specific reference values for NT-proBNP**

|  | **Males** | |  | **Females** | |
| --- | --- | --- | --- | --- | --- |
| **NT-proBNP (ng/L)** | **Upper reference value** | | | | |
| Age groups (years) |  |  | | |  |
| 0 - 44 | 86 |  | | | 130 |
| 45 - 55 | 121 |  | | | 249 |
| 55 - 65 | 210 |  | | | 287 |
| 65 - 75 | 376 |  | | | 301 |
| > 75 | 486 |  | | | 738 |

*Note:* Values represent the upper reference value of NT-proBNP per age group, derived from the local laboratory.

Abbreviation: NT-proBNP, N-terminal pro B-type natriuretic peptide.

**SUPPLEMENTAL TABLE 2 | Age- and sex-specific reference ranges for left ventricular mass index**

|  | **Males** | |  | | **Females** | | |
| --- | --- | --- | --- | --- | --- | --- | --- |
|  | **Bounds of the 95% prediction interval** | | | | | | |
| **LVMI (g/m^2^)** | **Lower** | **Upper** | |  | | **Lower** | **Upper** |
| Age groups (years) |  |  | |  | |  |  |
| 45 - 54 | 35 | 72 | |  | | 29 | 55 |
| 55 - 64 | 34 | 72 | |  | | 28 | 55 |
| 65 - 74 | 33 | 70 | |  | | 28 | 55 |

*Note:* Values represent the lower and upper reference limits of LVMI per age group, derived from the bounds of the 95% prediction interval. LVMI is calculated as the left ventricular mass divided by the body surface area, using the DuBois formula. Values are adapted from Petersen et al. 2017.

Abbreviation: LVMI, left ventricular mass index.

**SUPPLEMENTAL TABLE 3 | Age- and sex-specific reference ranges for estimated glomerular filtration rate**

|  | **Males** | |  | **Females** | |
| --- | --- | --- | --- | --- | --- |
|  | **Percentiles of the reference value** | | | | |
| **eGFR (mL/min/1.73/m^2^)** | **P2.5** | **P97.5** |  | **P2.5** | **P97.5** |
| Age groups (years) |  |  |  |  |  |
| 18 - 24 | 75 | 131 |  | 72 | 136 |
| 25 - 29 | 68 | 120 |  | 69 | 126 |
| 30 - 34 | 66 | 116 |  | 68 | 125 |
| 35 - 39 | 63 | 112 |  | 65 | 121 |
| 40 - 44 | 60 | 110 |  | 62 | 118 |
| 45 - 49 | 58 | 108 |  | 59 | 115 |
| 50 - 54 | 56 | 105 |  | 57 | 110 |
| 55 - 59 | 55 | 104 |  | 55 | 110 |
| 60 - 64 | 52 | 100 |  | 53 | 106 |
| 65 - 69 | 49 | 100 |  | 52 | 104 |
| 70 - 74 | 48 | 100 |  | 50 | 99 |
| 75 ≤ | 45 | 89 |  | 47 | 94 |

*Note:* Values correspond to the 2.5^th^ and 97.5^th^ percentiles of age-specific eGFR reference ranges. Values are adapted from Baba et al. 2015.

Abbreviation: eGFR, estimated glomerular filtration rate; P, percentile.
